# Supplementary material for: Light Stress in Yeasts: Signaling and Responses in Creatures of the Night
Source: Int J Mol Sci. 2023 Apr 8;24(8):6929. doi: 10.3390/ijms24086929 (PMC10139380; doi:10.3390/ijms24086929)
Supplement: Supplementary file 1 [file ijms-24-06929-s001.zip › Table S1 S cerevisiae genes R1.pdf]

Table S1 – *S. cerevisiae* genes involved in light response

Gene	Protein	Description[1]	Diffusion[2]
CRZ1	Transcription factor	Transcription factor, activates transcription of stress response genes; nuclear localization is positively regulated by calcineurin-mediated dephosphorylation; rapidly localizes to the nucleus under blue light stress; can be activated in stochastic pulses of nuclear localization in response to calcium	28
HAA1	Transcription factor	Transcriptional activator involved in adaptation to weak acid stress; activates transcription of *TPO2*, *YRO2*, and other genes encoding membrane stress proteins; relocalizes from cytoplasm to nucleus upon DNA replication stress	15
HEM15	Ferrochelatase	Ferrochelatase; a mitochondrial inner membrane protein, catalyzes insertion of ferrous iron into protoporphyrin IX, the final step in the heme biosynthetic pathway	30
MET8	Ferrochelatase	Bifunctional dehydrogenase and ferrochelatase; involved in the biosynthesis of siroheme, a prosthetic group used by sulfite reductase; required for sulfate assimilation and methionine biosynthesis	27
MGA2	Transcription factor	ER membrane protein involved in regulation of *OLE1* transcription; inactive ER form dimerizes and one subunit is then activated by ubiquitin/proteasome-dependent processing followed by nuclear targeting	29
MRH1	Rhodopsin	Protein that localizes primarily to the plasma membrane and the nuclear envelope; null mutation confers sensitivity to acetic acid	8
MSN2	Transcription factor	Stress-responsive transcriptional activator; activated in stochastic pulses of nuclear localization in response to various stress conditions; binds DNA at stress response elements of responsive genes; light sensing pathway component that accumulates in the nucleus in response to blue light; relative distribution to nucleus increases upon DNA replication stress	23
MSN4	Transcription factor	Stress-responsive transcriptional activator; activated in stochastic pulses of nuclear localization in response to various stress conditions; binds DNA at stress response elements of responsive genes, inducing gene expression; involved in diauxic shift	12
PHR1	Photolyase	DNA photolyase involved in photoreactivation; repairs pyrimidine dimers in the presence of visible light; induced by DNA damage; regulated by transcriptional repressor Rph1	21
POX1	FA-CoA Oxidase	Fatty-acyl coenzyme A oxidase; involved in the fatty acid beta-oxidation pathway; localized to the peroxisomal matrix; component of a light sensing pathway, converting light into a hydrogen peroxide signal, modulating nucleo-cytoplasmic shuttling of stress-responsive transcription factor Msn2	30
RPH1	Transcription factor	Repressor of autophagy-related genes in nutrient-replete conditions; damage-responsive repressor of *PHR1*; phosphorylated by the Rad53-dependent DNA damage checkpoint pathway and during starvation; target of stress-induced hormesis	29
YAP1	Transcription factor	Basic leucine zipper (bZIP) transcription factor; required for oxidative stress tolerance; activated by H_2O_2 through the multistep formation of disulfide bonds and transit from the cytoplasm to the nucleus; Yap1 is degraded in the nucleus after the oxidative stress has passed; mediates resistance to cadmium; relative distribution to the nucleus increases upon DNA replication stress	13

YRO2	Rhodopsin	Protein with a putative role in response to acid stress; null mutant is sensitive to acetic acid; transcription is regulated by Haa1p and induced in the presence of acetic acid; protein observed in plasma membrane foci in the presence of acetic acid	10

[1] Modified from SGD: gene overview/description (https://www.yeastgenome.org/).

[2] Results from GRYC database inquiry (http://gryc.inra.fr/index.php?page=home): total number of strains (out of 34 strains) containing at least one *locus*/gene with similarity (from weak to high) with the *S. cerevisiae* gene. High similarity scores were frequently found especially with *HEM15* and *POX1*. When a gene had an ohnolog in *S. cerevisiae*, the gene in the database could have been scored as similar to the ohnolog one. In this case the score was not registered in the Diffusion data column. Details of the inquiry are reported in **Table S2**.
