# Supplementary material for: Light Stress in Yeasts: Signaling and Responses in Creatures of the Night
Source: Int J Mol Sci. 2023 Apr 8;24(8):6929. doi: 10.3390/ijms24086929 (PMC10139380; doi:10.3390/ijms24086929)
Supplement: Supplementary file 1 [file ijms-24-06929-s001.zip › Table S2 Diffusion of genes R1.pdf]

Table S2 – Diffusion among yeasts of genes involved in light response

Gene (Sc)	Arad	Caal	Cagl	Capa	Cyfa	Deha	Ergo	Kana	Klla	Kuca	Laci	Lada	Lafa	Lafe	Lakl	Lala	Lame	Lami	Lano	Laqu	Lath	Lawa	Mifa	Naba	Nabr	Naca	Nade	Nani	Nuca	Tebl	Tomi	Yali	Zyba	Zyro	total
PHR1					1	1	1		1		1	1	1	1	1	1	1	1	1	1	1	1									1		1	2	19 (1)
YOR386W																							2										1		2 (1)
RPH1	1						1	2			1	1	1	1	1	1	1	1	1	1	1	1	2	1		1	1	1		2	1		1	1	24 (3)
YER169W			1		1	1			1																1										5 (0)
YRO2			2					1																2	1	2	2	1					1	1	9 (4)
YBR054W																																		1	1 (0)
MRH1											1			1	1			1							1			1			1				7 (0)
YDR033W	1																																		1 (0)
HAA1							1	2																						1					3 (1)
YPR008W	1		1			1			1										1					1	1	1	2	1					1	1	12 (1)
HEM15	1		1		1	1	1	1	1		1	1	1	1	1	1	1	1	1	1	1	1	2	1	1	1	1	1		1	1	1	1	1	30 (1)
YOR176W																																			
MET8			1					1	1		1	1	1	1	1	1	1	1	1	2	1	1		1	1		1	1		1	1		1	1	23 (1)
YBR213W	1				1	1																	2												4 (1)
MSN2							1	2																						2					3 (2)
YMR037C	1		1		1	3			1		1	1	1	1	1	1	1	1	1	1	1	1	4			1					1				20 (2)
MSN4							1	2																						2				1	4 (2)
YKL062W			2			1																			1	1	1	1				1	1		8 (1)
CRZ1							1	1																						1					3 (0)
YNL027W	1		1		3	1			1		1	1	1	1	1	1	1	1	1	1	1	1	2	1	1	1					1	1	1	1	25 (2)
POX1			1		1	2	1	1	1		1	1	1	1	1	1	1	1	1	1	1	1		1	1	1	1	1		1	1		1	1	27 (1)
YGL205W	1				1																		2												3 (1)
MGA2			1				1	2	1		1	1	1	1	1	1	1	1	1	1	1	1		1						1	1		1	1	21 (1)
YIR033W	1				1	1																			1	2	1	2				1			8 (2)
YAP1			1				1	2																						2					4 (2)
YML007W	1																						2	1	1	1	1	1					1	1	9 (1)

The first column reports the *S. cerevisiae* (Sc) gene names and the systematic annotations. The last column reports, in the upper position, the number of yeast strains with at least one *locus*/gene with similarity (similar or highly similar) with the *S. cerevisiae* gene. In the lower position, the number of strains with at least one *locus*/gene with weak or some similarity with the *S. cerevisiae* gene. In parentheses are reported the number of strains with two or more *loci* with similarity. The middle columns report the scores for each strain in the database (http://gryc.inra.fr/index.php?page=home). Columns are headed with the initials of the strain and are in alphabetical order. Upper and lower positions in each row refers to similar/highly similar scores and some/weak similarity scores, respectively. Strains are the following (modified from: http://gryc.inra.fr/index.php?page=home):
Arxula adeninivorans LS3; *Candida albicans* SC5314; *Candida glabrata* CBS 138; *Candida parapsilosis* CDC 317; *Cyberlindnera fabianii* YJS4271; *Debaryomyces hansenii* CBS 767; *Eremothecium gossypii* ATCC 10895; *Kazachstania naganishii* CBS 8797; *Kluyveromyces lactis* CBS 2359; *Kuraishia capsulata* CBS 1993; *Lachancea cidri* CBS 2950; *Lachancea dasiensis* CBS 10888; *Lachancea fantastica* CBS 6924; *Lachancea fermentati* CBS 6772; *Lachancea kluyveri* CBS 3082; *Lachancea lanzarotensis* CBS 12615; *Lachancea meyersii* CBS 8951; *Lachancea mirantina* CBS 11717; *Lachancea nothofagi* CBS 11611; *Lachancea quebecensis* CBS 14088 ; *Lachancea thermotolerans* CBS 6340; *Lachancea waltii* CBS 6430; *Millerozyma farinosa* CBS 7064; *Nakaseomyces bacillisporus* CBS 7720; *Nakaseomyces bracarensis* CBS 10154; *Nakaseomyces castellii* CBS 4332; *Nakaseomyces delphensis* CBS 2170; *Nakaseomyces nivariensis* CBS 9983; *Naumovozyma castellii* CBS 4309; *Tetrapisispora blattae* CBS 6284; *Torulaspora microellipsoides* CLIB830; *Yarrowia lipolytica* H222, E150, A101; *Zygosaccharomyces bailii* CBS 680; *Zygosaccharomyces rouxii* CBS 732.
